# Helper T Cells are Hyperactive and Contribute to the Dysregulation of Antibody Production in Patients with Rheumatoid Arthritis

**DOI:** 10.3390/ijms251810190

**Published:** 2024-09-23

**Authors:** Mustafa Talib, Balázs Gyebrovszki, Dorottya Bőgér, Réka Csomor, Anna Mészáros, Anna Fodor, Bernadette Rojkovich, Gabriella Sármay

**Affiliations:** 1 Department of Immunology, Eötvös Loránd University, 1053 Budapest, Hungary; talibmustafa19@gmail.com (M.T.); gyebrovszki.balazs@gmail.com (B.G.); boger.dorottya@gmail.com (D.B.); cs.reka19@gmail.com (R.C.); annameszaros5@gmail.com (A.M.); fodor.anna94@gmail.com (A.F.); 2 Rheumatology-Rehabilitation Department, Buda Hospital of the Hospitaller Order of Saint John of God, 1027 Budapest, Hungary; rojkovich.b@gmail.com

**Keywords:** B-cell differentiation, IgG production, IL-21, PD-1, Th cells, Treg cells

## Abstract

Rheumatoid arthritis (RA) is a systemic autoimmune disease, mediated by a complex interaction between B cells and various subsets of T cells. Dysfunction of helper T (Th) and regulatory T (Treg) cells may contribute to the breakdown of self-tolerance and the progression of autoimmune disease. In this study, we investigated the activity of Th and Treg cells on the differentiation of autologous B cells in vitro using cell cultures from the peripheral blood of healthy controls (HCs) and RA patients. The expressions of programmed death 1 (PD-1) and IL-21 were monitored as activation markers for Th cells. Unstimulated Th cells from RA patients showed remarkably higher PD-1 expression than HC samples. Stimulation of Th cells from RA patients with Staphylococcus enterotoxin B (SEB) in the presence of B cells significantly induced their PD-1 and IL-21 expression at a considerably higher level in RA compared to HCs, and Treg cells did not affect IL-21 production. When monitoring B-cell differentiation, a significantly higher frequency of plasma cells was observed, even in unstimulated samples of RA patients compared to HCs. In the SEB-stimulated co-cultures of the RA samples, plasma cell frequency and IgG production were considerably higher than in HCs and were not significantly affected by Tregs. These findings demonstrate that Th cells are constitutively active in RA, and their hyperactivity upon interaction with diseased B cells may lead to uncontrolled antibody production.

## 1. Introduction

Rheumatoid arthritis (RA) is a chronic inflammatory autoimmune disease with unknown specific causes, characterized by synovial inflammation and autoantibody production, which leads to cartilage and bone destruction and disabilities [1,2,3]. Several factors, such as genetic predisposition, environmental influences, gender, and life habits, contribute to the development of RA by dysregulating immune tolerance [3,4,5]. The chronic inflammation in RA is caused by the overproduction of various proinflammatory cytokines, which results in an excessive activation of T and B cells, with an uncontrolled production of autoantibodies targeting self-antigens or post-translationally modified molecules, such as rheumatoid factor (RF) or anti-citrullinated protein antibodies (ACPA) [4,6,7], that are involved in RA pathogenesis [8].

The autoimmune response may be driven by elevated type 1 helper T cell (Th1) cell activation, accompanied by insufficient type 2 helper T cell (Th2) cell differentiation, which ensures the persistence of the pro-inflammatory microenvironment in the synovium by the secretion of TNFα, IFNγ, and IL-2, resulting in cartilage destruction and bone erosion [9,10,11,12]. IL-2 regulates the differentiation and development of both Tregs and Th17 cells [13,14,15]. However, the number of Tregs negatively correlated with disease activity in autoimmune diseases, indicating that the disruption of immune tolerance by the decrease of Tregs might be crucial in RA pathogenesis [16,17,18]. Th17 cells enhance bone destruction by inducing receptor activators of nuclear factor kappa beta (NF-kβ) ligand (RANKL) expression on osteoclasts through IL-17 and IL-23 [11,12].

Uncontrolled activation of CD4^+^Th cells in RA also leads to the expansion of autoreactive follicular T helper (Tfh) cells and peripheral T helper (Tph) cells [19,20]. Tfh cells are indispensable for germinal center formation and autoantibody-producing B-cell development by releasing IL-21 and CXCL13 [19,21,22]. Similarly, Tph cells, which are characterized as PD-1^hi^CXCR5^–^CD4^+^T cells, uniquely express B-lymphocyte-induced maturation protein 1 (BLIMP1) and chemokine receptors (CCR5, CX3CR1, and CCR2) that enhance their migration into inflammatory sites. Functionally, Tph cells resemble Tfh cells, and both contribute to RA pathogenesis by inducing plasma cell differentiation through IL-21 secretion [23,24]. Circulating counterparts of both Tfh and Tph cells, albeit in small numbers, can be detected and analyzed in the peripheral blood of RA patients [25,26,27].

Th and Treg cells have opposite functions. Tregs suppress various Th-cell-mediated proinflammatory immune responses and participate in maintaining self-tolerance by constitutively expressing Foxp3 and by releasing IL-10 and TGF-β upon activation, as well as by expressing inhibitory receptor CTLA-4, TIGIT, and PD-1 molecules [17,18]. Currently, cells with the CD3^+^CD4^+^CD25^high^CD127^low^ phenotype are considered human Tregs. Contradictory results were reported for the functional characteristics and number of Treg cells in RA patients [17].

Overall, various CD4^+^T cell subsets were shown to significantly contribute to RA pathogenesis by producing pro-inflammatory cytokines and promoting autoantibody production. However, the functional activity of the circulating Th and Treg cells within the same cohort of patients has not been investigated. Therefore, in the current study, we isolated CD4^+^CD25^low/−^CD127^hi^Th/cTfh/Tph cells and CD4^+^CD25^high^CD127^low^Treg/cTfr cells from the peripheral blood of RA patients and HCs and compared the activation state of the Th cells by monitoring PD-1 expression and IL-21 production. Further, we monitored the same parameters in co-cultures of Th and B cells activated with Staphylococcus enterotoxin B (SEB) superantigen, which activates T cells by crosslinking TCR and CD28 on T cells with MHC-II and B7 molecules on B cells [28] This system closely mimics the physiological situation in which Tfh cells support B-cell activation and antibody production. Additionally, we investigated to what extent Treg cells would influence Th-cell-dependent B-cell stimulation in both cases.

## 2. Result

### 2.1. Characterization and Sorting of the CD4^+^ T Cell Subsets for Functional Studies

**Figure 1:**
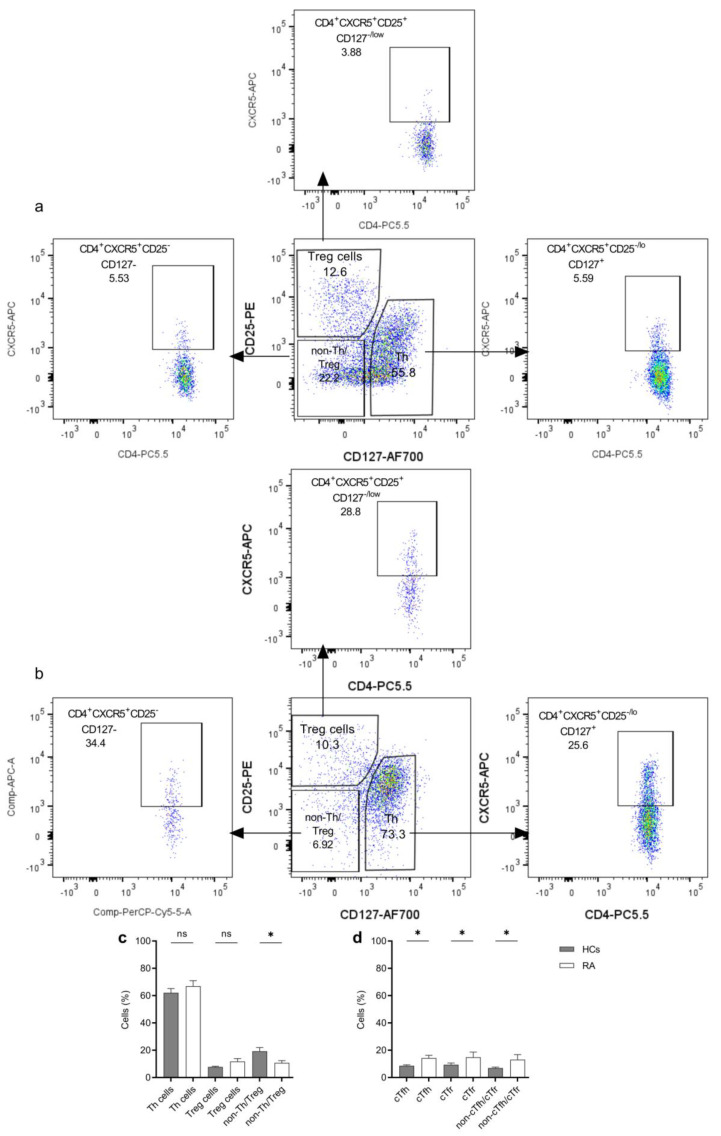
Gating strategy and frequency of CD4^+^T cell subsets. (**a**) and (**b**) Representative gating strategies of CD4^+^T cell subsets from HCs and RA patients, respectively. (**c**) Frequency of the Th, Treg, and non-Th/Treg cells among CD4^+^T cells from HCs and RA patients. (**d**) Frequency of the CXCR5 positive cTfh, cTfr, and non-cTfh/cTfr cells among Th, Treg, and non-Th/Treg cells, respectively, from HCs (*n* = 20) and RA patients (*n* = 9). The quantification is presented as mean ± S.E.M, using ordinary one-way ANOVA with uncorrected Fisher’s LSD. The significance was taken as * *p* < 0.5. ns: not significant, *Treg cells*; regulatory T cells, *Th cell*; helper T cells, *cTfh*; circulatory follicular helper T cells, *cTfr*; circulatory follicular regulatory T cells.

We measured the frequency of the Th cells (CD4^+^CD127^+^CD25^−/lo^), Treg cells (CD4^+^CD127^−/lo^CD25^+^), and non-Th/Treg cells (CD4^+^CD127^-^CD25^−^), and within these subsets, we also evaluated the frequency of circulating follicular helper T (cTfh) cells (CD4^+^CXCR5^+^CD127^+^CD25^−/lo^), circulating follicular regulatory T (cTfr) cells (CD4^+^CXCR5^+^CD127^−/lo^CD25^+^) and non-cTfh/cTfr cells (CD4^+^CXCR5^+^CD127^−^CD25^−^), respectively, among the HCs and RA patients (Figure 1a,b and Figure S1a,b). The frequency of Th and Treg cells was similar between the two groups, while the non-Th/Treg cells were significantly higher in HCs (Figure 1c).

To identify the circulating Tfh and Tfr subsets, we included CXCR5, the defining marker for follicular T cells, into the antibody panel for flow cytometry. The CXCR5^+^ cTfh, cTfr, and non-cTfh/cTfr cells were present in both HC and RA patients’ samples, and all circulating CD4^+^CXCR5^+^ T-cell subsets have shown a significantly higher frequency in RA compared to HCs (Figure 1d). However, the number of cells was not sufficient for sorting them to carry out functional assays. Therefore, we sorted the Th and Treg cell subsets from RA patients and HC to monitor their functional activities in SEB-stimulated co-cultures with autologous B cells. The gating strategy for sorting is shown in Figure 1a,b. To validate the gating, both the Th and Treg subsets were checked for Foxp3 expression, and indeed, the majority of the CD4^+^CD127^low/−^CD25^hi^ cells have shown a high staining intensity with anti-Foxp3, thus corresponding to Treg cells (Figure S1a).

### 2.2. Helper T Cells from RA Patients Are Hyperactive

**Figure 2:**
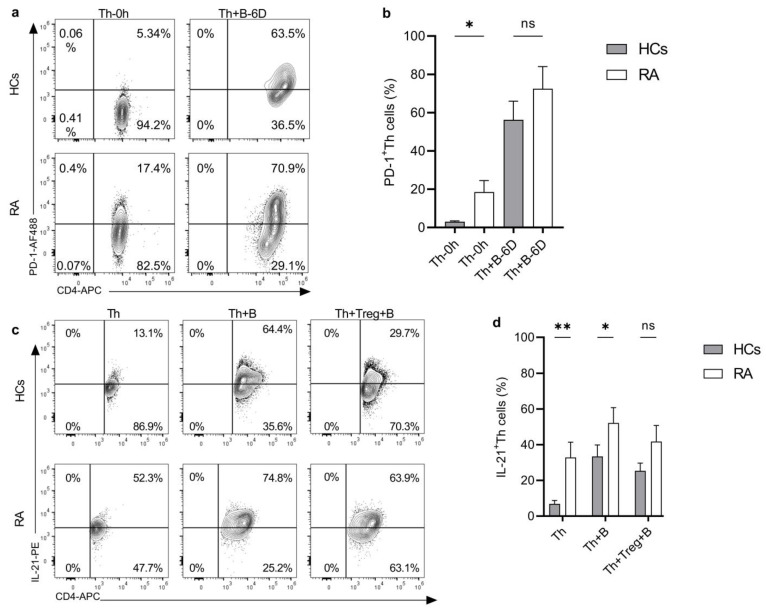
Differential expression of PD-1 and IL-21 on Th cells from healthy individuals and RA patients. (**a**) Representative example of PD-1 expression on sorted CD4^+^CD25^-^CD127^+^ Th cells isolated from peripheral blood of HCs and RA patients before (left panels) and after (right panels) stimulation with SEB for 6 days in coculture with B cells (Th+B). Numbers within each quadrant represent the percentage of PD-1^+^ Th cells in a representative experiment. (**b**) Quantification of PD-1^+^ Th cells out of independent experiments; unstimulated Th cells (*n* = 8) and Th+B cell co-cultures from HCs (*n* = 10); and unstimulated Th cell (*n* = 6) and Th+B cells co-cultures (*n* = 3) from RA patients. (**c**) Evaluation of a representative experiment for IL-21 production by Th cells in SEB stimulated monoculture (Th), Th+B cell coculture, and in presence of Treg cells (B+Th+Treg) from healthy donors (upper panel) and RA patients (lower panel). (**d**) Quantification of IL-21 producing Th cells from independent experiments from HCs: Th monocultures (*n* = 9), B+Th (*n* = 9), and B+Th+Treg (*n* = 7). And from RA patients, Th monocultures (*n* = 7), B+Th (*n* = 8), and B+Th+Treg co-cultures (*n* = 6). All culture systems were prepared by seeding 5 × 10^4^ Th cells/well in monocultures and mixed at a 1:1 ratio with B cells and Treg cells in co-culture and triple culture and stimulated for six days using SEB superantigen (22 ng/mL). The quantification is presented as mean ± S.E.M, using ordinary one-way ANOVA with uncorrected Fisher’s LSD. The significance was taken as * *p* < 0.5, ** *p* < 0.01. ns: not significant.

In recent years, it has been demonstrated that the PD-1 expression on the peripheral blood T cells of RA patients positively correlates with the disease activity score [29]. Furthermore, a significantly elevated number of circulating PD-1^hi^CD4^+^T cells that promote B-cell differentiation via secreting IL-21 were detected in RA patients compared to HCs. [30]. Therefore, we investigated the basal level of PD-1 expression on freshly isolated Th cells and in SEB-stimulated Th cells from the co-cultures with autologous B cells. In concert with previous findings, we have found a significantly higher PD-1 expression on Th cells from RA patients compared to HCs, and this was significantly upregulated in SEB-stimulated co-cultures with B cells in both groups (Figure 2a,b and Figure S2). Next, we evaluated the intracellular IL-21 production by Th cells in the same setting, after 6 days of stimulation with SEB alone, and in a co-culture with B cells. An example of full gating is shown in Figure S3. We have found a significantly elevated frequency of IL-21-producing Th cells in RA patients’ samples (32.8% ± 8.6%) compared to healthy individuals (6.9% ± 1.96%), indicating a constitutive IL-21 production in RA (Figure 2c,d). In the co-cultures (Th+B cell), IL-21 production was upregulated in both HCs and RA patients’ samples, with 33.41% ± 6.5% and 52.2% ± 8.5%, respectively, and by comparing the two groups, Th cells from RA patients showed a significantly higher IL-21 production compared to HCs. (Figure 2c,d). Additionally, we evaluated the IL-21 expression in the presence of cognate Treg cells in triple cultures. IL-21 production tends to be downregulated by Treg cells in both HCs and RA patients’ samples, but no significant differences were observed (Figure 2c,d). Thus, Th cells from RA patients are highly active and more susceptible to stimulation, resulting in a higher level of IL-21 production compared to HCs, while Treg cells from both groups did not significantly reduce the IL-21 production.

### 2.3. Th Cells Induce a Similar Rate of B-Cell Proliferation in Co-Cultures from Both Healthy Controls and RA Patients

**Figure 3:**
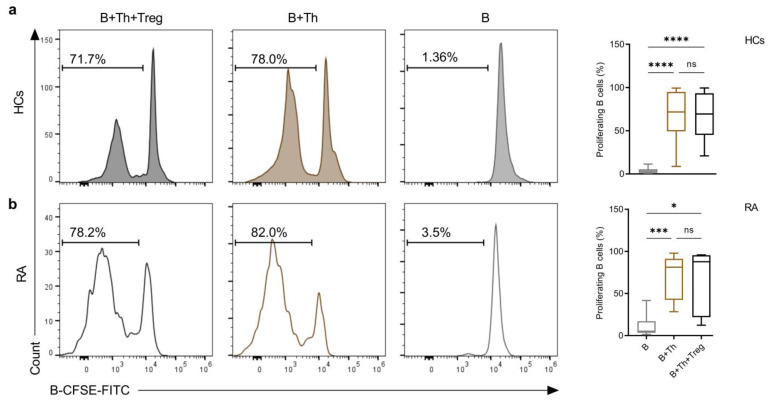
Th-cell-induced proliferation of B cells from HCs (**a**) and RA (**b**) patients. (**a**) and (**b**) Histograms depict a flow cytometric analysis of B-cell proliferation from HCs and RA patients, respectively. Cells were uploaded with CFSE dye and stimulated in monoculture (B), co-culture (B+Th), or triple culture (B+Th+Treg) for six days in the presence of SEB superantigen (22 ng/mL). The bar charts show the evaluation of the results from at least (*n* = 5) independent experiments using ordinary one-way ANOVA with uncorrected Fisher’s LSD. The significance was taken as * *p* < 0.5, *** *p* < 0.001, **** *p* < 0.0001. ns: not significant.

Th cells induce B-cell proliferation, differentiation, and antibody production upon the T-dependent immune response. Therefore, we investigated the proliferation efficacy of B cells in SEB-stimulated co-cultures with Th cells in the absence and in the presence of Treg cells. B cells from both groups were uploaded with CFSE dye before stimulation, and the percentage of proliferating cells was monitored in HCs and RA samples by following the dilution out of the CFSE in B cells As expected, the percentage of proliferating B cells highly increased in co-cultures compared to B cell monocultures of both groups, but no difference was observed between HCs and RA patients (Figure 3a,b, full gating strategy Figure S4). Treg cells in triple cultures did not seem to influence B cell proliferation in both groups (Figure 3a,b).

### 2.4. Th Cells from RA Patients Induce a Higher Degree of Plasma Cell Differentiation in Th+B Cell Co-Cultures Compared to HCs

**Figure 4:**
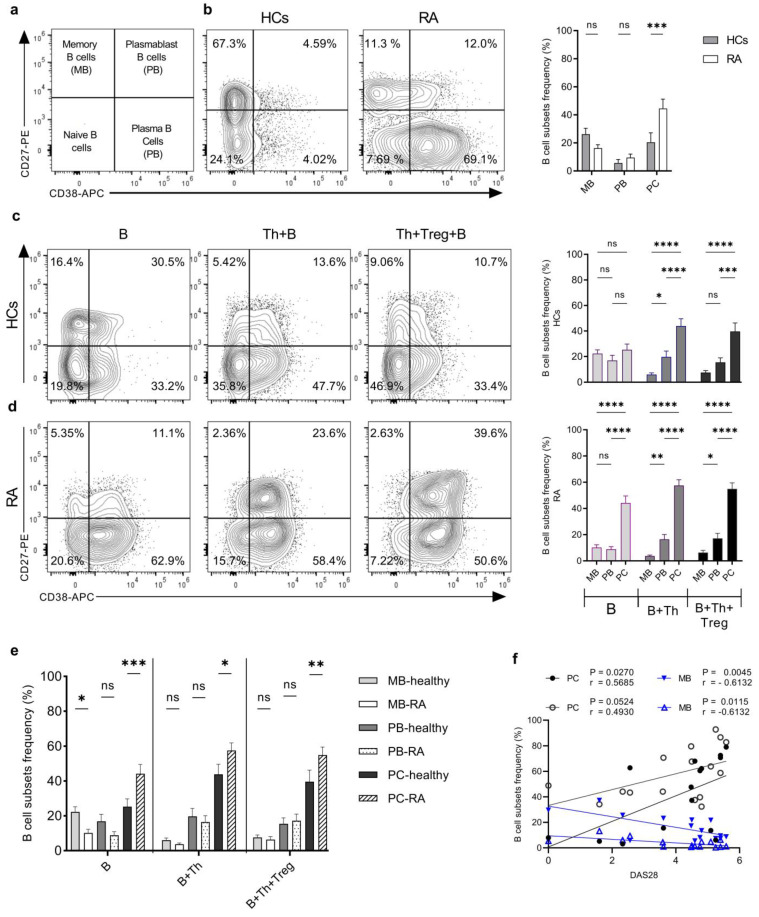
Th cells from RA patients induced differentiation and plasma cell generation from autologous B cells at a higher rate as compared to HCs. (**a**) Illustration of B-cell subsets, memory B cells (MB), plasma blast (PB), and plasma cells (PC) distribution in different quadrants according to CD19, CD27, and CD38 expression. (**b**) A representative example of flow cytometric analysis of the distribution of B-cell subsets, within freshly isolated CD19^+^ B cells from the blood of HCs and RA patients. The quantifications in the bar chart show the averages from (*n* = 15) independent experiments. (**c**), Flow cytometric analysis of the distribution of B cell subsets from HCs’ and (**d**) RA patients’ samples, stimulated with SEB for 6 days in monoculture (B), co-culture with Th cells (B+Th), and in the presence of Treg cells (B+Th+Treg). Flow cytometric analysis of a representative experiment (left panels) and quantification of B-cell subset frequencies (bar charts) are shown: (HCs-MB; *n* = 19, PB; *n* = 19, PC; *n* = 16) and (RA-MB; *n* = 16, PB; *n* = 16, PC; *n* = 14). (**e**) Accumulative bar chart comparing the distribution of MB, PB, and PC subsets between HCs and RA patients within the same culture systems. The number of cells per subset was 5 × 10^4^ cells, and a 1:1 ratio was applied in the cases of coculture and triple culture. The statistical model ordinary two-way ANOVA with uncorrected Fisher’s LSD was used and the significance was evaluated as * *p* < 0.5, ** *p* < 0.01, *** *p* < 0.001, **** *p* < 0.0001. ns: not significant. (**f**) Correlation between the frequencies of plasma cells and memory B cells, respectively, and the disease activity score (DAS 28) of RA patients, the two upward lines correspond to plasma cells (PC) before stimulation (black filled circle) and after stimulation (black open circle). The two downward lines represent memory B cells (MB) before stimulation (blue filled triangle) and after stimulation (blue open triangle).

To determine how activated Th cells influence the differentiation of B cells into plasma cells, we screened B cells for CD19, CD27, and CD38 expression to identify memory B cells, plasma blasts, and plasma cells (Figure S5). Before stimulation, the level of CD19^+^CD27^+^CD38^−^ memory B cells was somewhat higher in HC samples, and we detected a significantly higher frequency of CD19^+^CD27^−^CD38^+^ plasma cells in RA patients’ samples (44.2% ± 5.3%) compared to HCs (25.3% ± 4.5%) (Figure 4b). On the 6th day, there was no significant difference between B-cell subsets in monocultures of B cells in the case of HCs (Figure 4c). In contrast, plasma cells were the most prominent subset in B-cell monocultures from RA patients (Figure 4d). In the Th+B cell co-cultures, activated Th cells significantly upregulated both plasma blasts and plasma cell differentiation in both groups (Figure 4c,d). Treg cells were able to significantly modulate the ratio of plasma blasts only in HCs (Figure 4c), whereas they did not affect the differentiation of B cells from RA patients (Figure 4d). However, when we compared the subsets of B cells in co-cultures between HCs and RA patients, we found that plasma cells were the most frequent cells in RA patient samples in all culture conditions, and their frequency was significantly higher compared to HCs (Figure 4e). Furthermore, the percentage of plasma cells was positively correlated with DAS28 values, both before and after stimulation with Th cells, whereas the percentage of memory cells showed a negative correlation (Figure 4f).

### 2.5. Helper T Cells from RA Patients Induced a Higher IgG Production by Plasma Cells Compared to Healthy Samples

**Figure 5:**
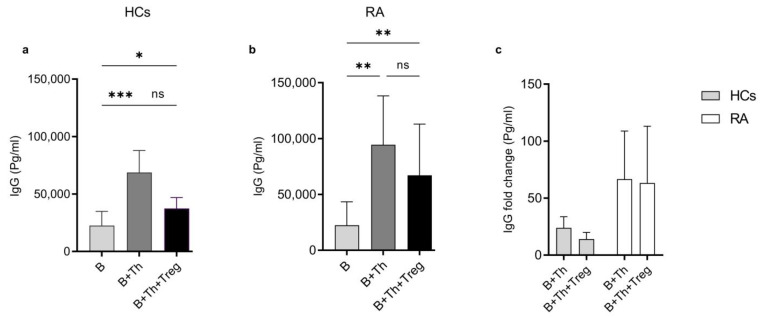
Activated Th cells from RA patients (**b**,**c**) induce higher IgG production when cocultured with autologous B cells as compared to HCs (**a**,**c**). IgG production was evaluated from the harvested supernatant of (B), (B+Th), and (B+Th+Treg) cell cultures by ELISA. IgG produced by B cells in monoculture was used to calculate the fold change. All culture systems were prepared as in (Figure 4). The quantification was applied for (*n* = 21) independent measurements for HCs and (*n* = 14) for RA patients (mean % ± S.E.M.). The Kruskal–Wallis test with uncorrected Dunn’s test was used for evaluation * *p* < 0.5, ** *p* < 0.01, *** *p* < 0.001. ns: not significant.

As shown in Figure 4, Th cells induce a higher rate of plasma cell differentiation in RA patients’ samples compared to HCs. Plasma cells significantly contribute to RA pathogenesis by producing autoantibodies. Consequently, we evaluated IgG production from the harvested culture supernatants of the corresponding culture systems from both types of donors. As expected, Th cells promoted IgG secretion by B cells in both groups, but the enhancement was higher in RA patients’ samples (Figure 5a,b). When Treg cells were introduced in triple cultures, IgG synthesis was slightly reduced in HCs and less efficiently in RA patients’ samples (Figure 5c). These results together indicate that B cells from RA patients produce higher levels of IgG in the in vitro culture with Th cells compared to B cells from HCs, mainly due to the hyperactivity of the Th cells, and Treg cells are more effective in downregulating IgG synthesis in HC samples.

## 3. Discussion

The mechanism of B-cell regulation by Th-cell subsets has been extensively studied [10,19,31,32,33,34]. Within the germinal centers of secondary lymphoid organs, B-cell activation is orchestrated by follicular helper T (Tfh) and follicular regulatory T (Tfr) subsets [19,35,36]. In the periphery, however, their circulating counterparts, namely cTfh/Tph, and cTfr cells, are the main players in the regulation [37]. In RA and various inflammatory autoimmune diseases, dysregulation of the number and/or function of these cells has been shown to be associated with autoantibody production, as well as with disease progression [19,37]. Indeed, dysregulation of other Th-cell subpopulations, defined as Th1, Th17, Th22, Th9, and Treg cells, directly or indirectly contribute to RA pathogenesis by influencing B-cell activation [10,38]. Yet, more remains to be elucidated about the mechanism of Th-cell-dependent B-cell activation in RA patients [22,25,39]. Functional studies based on cTfh, Tph, and cTfr subsets are limited due to their low frequency in the blood. Therefore, we have used Th and Treg cells separated from the peripheral blood of RA patients and HCs based on their differential expression of CD25 and CD127 and compared their activity on B-cell differentiation. We have identified constitutively active Th cells from the blood of RA patients that expressed PD-1, produced high levels of IL-21, and drastically induced B-cell activation by enhancing their differentiation and antibody production. In terms of their function, these cells resemble cTfh and/or the recently characterized cTph cells, which have been shown to play a crucial role in inflammatory autoimmune diseases by providing help for B cells in the periphery for local antibody production [30,40,41].

The investigation of PD-1 expression on unstimulated and IL-21 production by SEB-stimulated Th cells in a monoculture led to the identification of PD-1/IL-21 highly expressing Th cells in RA patients, representing a pre-activation state of Th cells. We hypothesized that PD-1^hi^/IL-21-producing cells might have an enhanced B-cell activation effect. Indeed, the in vitro stimulation showed a significant upregulation of both PD-1 and IL-21 expression by Th cells in the co-culture with autologous B cells, with a higher proportion in RA samples than in HCs, confirming the pre-activated state of the former. On the other hand, SEB alone was able to induce a significant IL-21 release in the monoculture of Th cells from RA patients, which could be explained by the abundant expression of TCR and CD28, as well as HLA-DR antigen, on these cells [42], which would increase the sensitivity to TCR-mediated signals, even in the absence of co-stimulation by B cells [43].

The increased percentage of IL-21^+^ Th cells after stimulation in co-cultures of both groups studied with a higher proportion in the RA patients’ samples suggests that the majority of Th cells might have gained a B-helper T-cell phenotype, which is predominantly characterized by the expression of IL-21 [24,44]. Although SEB-activated Th cells from RA patients were highly active and produced high levels of IL-21, they induced a similar rate of B-cell proliferation compared to HCs. Furthermore, Treg cells from both RA patients and HCs were unable to suppress B-cell proliferation, suggesting that Tregs do not play a role in this process.

The contribution of B cells to the pathogenesis of RA has been extensively studied, and the production of autoantibodies, such as ACPAs and RF, has been described [4,7,45,46]. Here, we characterized the phenotypes of B cells in SEB-stimulated co-cultures with Th cells from HCs and RA patients and demonstrated that antibody-producing plasma cells were the most frequent cell type in RA samples, even before stimulation. And their number was positively correlated with disease activity, whereas memory B cells were present in higher proportions in the HC samples and were negatively correlated with disease activity in RA patients. Notably, a similar pattern of correlation was observed before and after stimulation in co-cultures with Th cells. This finding illustrates the constitutively activated state of B cells in RA patients, which is consistent with the activated state of Th cells from the same group.

B cells contribute to the pathogenesis of RA by promoting Tfh and Tph cell generation by presenting autoantigens to Th cells, thereby inducing autoreactive B-cell differentiation into plasma cells, which is the major source of autoantibodies such as ACPAs and RF [38,45,46]. We showed that, after the in vitro activation of B cells in co-culture with IL-21-producing Th cells, a significantly higher rate of plasma cell differentiation can be detected in samples from RA patients compared to HC, most likely due to the higher level of IL-21. In addition, Treg cells were unable to suppress B-cell differentiation in both RA and HC samples.

In line with the elevated frequency of plasma cells in RA patients, we detected a significantly higher level of IgG production by B cells in co-cultures with autologous Th cells from RA patients compared to HCs. Remarkably, IgG production was reduced when Treg cells were introduced into the co-cultures in both the RA and HC samples, although the differences were not found to be significant. However, the reduction was greater in healthy controls than in RA patients’ samples, indicating a high activation status of Th cells and an impaired Treg function in RA patients.

In conclusion, we have shown here that Th cells, most likely corresponding to cTfh and/or cTph cells from RA patients, are characterized by an enhanced activation status compared to HCs. Accordingly, these cells are PD-1+ and produce higher levels of IL-21, which induces B-cell differentiation into plasma cells and significantly increases IgG production. On the other hand, the regulatory function of Treg cells may be impaired in RA patients, representing a dysfunctional phenotype that, together with the hyperactivity of Th cells, may contribute to the induction of autoantibodies and, thus, to the pathogenesis of RA.

Although the current study investigated the effect of Th cells on B cells by considering only IL-21 production, further cytokines need to be tested in future studies to gain more insight into the contribution of other cytokines to B-cell activation and differentiation in RA patients. In addition, a larger number of patients in future studies would significantly improve the current findings by minimizing the variance between donors.

## 4. Materials and Methods

### 4.1. Blood Samples

Human peripheral blood samples were obtained from individuals diagnosed with RA according to the revised classification criteria of the American College of Rheumatology/European League Against Rheumatism (ACR/EULAR) [1] and from age and sex-matched healthy controls who had not been vaccinated in the last three months and had no inflammatory or autoimmune disease. The clinical and demographic data of RA patients are summarized in Table 1.

The blood samples were taken into lithium heparin-containing VACUETTE^®^ TUBEs (Greiner Bio-one) after a written informed consent was signed. The study was conducted by following the Declaration of Helsinki. The patients recruited for this study might be treated with conventional disease-modifying antirheumatic drugs, such as methotrexate and/or Medrol, but they have not received biological therapy for three months before the blood samples were taken.

The study was ethically approved by the Scientific and Research Ethics Committee of the Health Science Council and the National Center for Public Health and Pharmacy (NCPHP) (42578-6-2018/EÜIG).

### 4.2. Isolation of B and T Cells Using Magnetic Activating Cell Sorting (MACS)

Peripheral blood mononuclear cells (PBMCs) were extracted from approximately 50 mL of blood samples immediately after collection via density gradient centrifugation using a PBMC Spin Medium (PluriSelect, Leipzig, Germany). The peripheral blood was diluted with 2 µM EDTA containing phosphate-buffered saline (PBS) in a 1:2 ratio. Then, 30 mL of diluted blood was layered on 15 mL of PBMC Spin Medium in Leucosep tubes (Greiner Bio-one, Kremsmünster, Austria) and centrifuged for 15 min at 800× *g* at room temperature (RT) using a swing-out bucket rotor with soft stop brakes. PBMCs were collected by transferring the suspension above the porous barrier to a new 50 mL falcon tube and washed twice with 2 µM EDTA-PBS. Finally, the PBMCs were counted and filled with 50 mL of complete RPMI-1640 medium (10% FCS, RPMI-1640 with L-glutamine, 1% streptomycin/penicillin (Sigma-Aldrich, St. Louis, MO, USA) and stored at 4 °C overnight.

T and B cells were negatively selected by using the Pan T cell (Lot No. 5220205924) and Pan B cell (Lot No. 5211003043) isolation kits, respectively, and LS columns, all from Miltenyi Biotec. PBMCs were washed with MACS buffer (0.5% BSA, 2 µM EDTA in PBS, pH 7.4) for 10 min at 300× *g* at 4 °C, and the reagents were used following the manufacturer’s guidance. The purity was checked using Brilliant Violet 421™ conjugated anti-human CD19 mAbs, BV421™ (Clone: HIB19, BioLegend, San Diego, CA, USA); phycoerythrin (PE)-conjugated anti-human CD3 mAbs (Clone: UCHT1, BioLegend), and PerCp/Cyanine5.5 anti-human CD4 (PC5.5): Clone. RPA-T4, (5 µg/mL) for B and T cells, respectively. The list of antibody conjugates and isotype controls used throughout the study is shown in Table S1.

### 4.3. Isolation of Th Cells and Treg Cells Using Fluorescence-Activated Cell Sorting

After being isolated from PBMCs by MACS, the T cells were stained with PerCp/Cyanine5.5 anti-human CD4 (PC5.5): Clone. RPA-T4, (5 µg/mL); phycoerythrin (PE) anti-human CD25: Clone. BC96, (2.5 µg/mL); and Alexa Fluor^®^700 anti-human CD127 (AF700): Clone. A019D5, (10 µg/mL). The Th cells were identified as CD4^+^CD127^+^CD25^−/lo^ T cells, and the Treg cells were identified based on expressing CD4^+^CD127^−/lo^CD25^hi^ using a BD FACSAria™ III Cell Sorter. Additionally, we used Alexa Fluor^®^647 anti-human CD185 (CXCR5): Clone. J252D4, (5 µg/mL) to characterize the circulating follicular helper T (cTfh) cells (CD4^+^CXCR5^+^CD127^+^CD25^-/lo^), circulating follicular regulatory T (cTfr) cells (CD4^+^CXCR5^+^CD127^−/lo^CD25^+^), and non-cTfh/cTfr cells (CD4^+^CXCR5^+^CD127^−^CD25^−^). Stained and fluorescent minus one (FMO) samples were applied for compensation controls and the appropriate setting of the gates. T cells were blocked with 10% mouse serum (MS) for 20 min, and the staining procedure was applied on ice and protected from light. After the last washing step, the cells were resuspended in 5% FCS-PBS and filtered through a 70 µm strainer for sorting. All procedures were conducted under sterile conditions. Immediately after sorting, the Th cells and Treg cells were evaluated for Foxp3 expression using True-Nuclear™ Transcription Factor kits (BioLegend) with PE anti-human Foxp3 monoclonal antibody (Clone. QA18A03 BioLegend) (2.5 µg/mL).

### 4.4. Immunophenotyping Assay

B-cell subsets (memory B cells, MB; plasma blast, PB; and plasma B cells, PC) were evaluated from freshly isolated B cells after being blocked with 10% MS for 20 min and stained with anti-CD19-BV421™, PE-conjugated anti-human CD27 monoclonal antibody (Clone: O323, BioLegend; 2.5 µg/mL), and allophycocyanin (APC) conjugated anti-human CD38 monoclonal antibody (Clone: HIT2, ImmunoTools, Friesoythe, Germany; 5 µL) using CytoFLEX LS flow cytometry (Beckman Coulter, Brea, CA, USA), and 7-Aminoactinomycin D (7AAD) was used to exclude the dead cells from the analysis. MB was characterized as CD19^+^CD27^+^CD38^−^ B cells, PB was defined as CD19^+^CD27^+^CD38^+^ B cells, and PC was specified as CD19^+^CD27^−^CD38^+^ B cells. We set different culture systems: Th cells monoculture (Th), B cell monoculture (B), B cells with Th cells (co-culture, B+Th), and B cells with Th and Treg cells (triple culture, B+Th+Treg). The isolated cells (B cells, Th, and Treg cells) were adjusted to 10^6^ cells/mL using complete RPMI-1640, and then, we distributed the cells into 96-well culture plates (TC plate 96 well, SARSTEDT AG & Co. KG, Nümbrecht, Germany). For the monoculture, 5 × 10^4^ cells were used, and a ratio of 1:1 was applied for co- and triple cultures. Finally, the cultures were stimulated with Staphylococcus enterotoxin B (SEB) (22 ng/mL) in a final volume of 200 µL complete RPMI-1640 for 6 days. At the end of the incubation period, all cell cultures were harvested into round-bottom polystyrene 5 mL tubes and centrifuged (300× *g*, 4 °C, 5 min), and the supernatants were collected and stored at −70 °C for the assessment of IgG production. The remaining cultured cells were used for phenotyping B-cell subsets and measuring IL-21 production by Th cells. IL-21 was evaluated by intracellular fluorescence in Th, B+Th, and B+Th+Treg cell cultures after being stimulated for an additional 5 h with 50 ng/mL Phorbol 12-myristate 13-acetate (PMA) (Sigma-Aldrich); 1 µg/mL Ionomycin (Sigma-Aldrich); and BD GolgiPlug™ protein transporter inhibitor containing Brefeldin A; 1:1000 (Becton-Dickinson, Franklin Lakes, NJ, USA). The cells were washed, blocked with 10% MS, and stained for extracellular marker CD4 using either anti-CD4-PC5.5 or anti-human CD4-APC (Clone: EDU-2, ImmunoTools; 5 µL), and PE conjugated anti-human IL-21 monoclonal antibody (Clone: 3A3-N2, BioLegend; 2.5 mg/mL) was applied after the fixation and permeabilization steps, which were performed using BD Cytofix/cytopermTM Plus Fixation/Permeabilization kits (Becton-Dickinson), as instructed by the vendor. To implement gating control, a sample was stained with the relevant isotype control for each panel that was developed.

### 4.5. Proliferation Assay

We assessed B-cell proliferation in the specified culture systems used for the immunophenotyping assay. B cells were uploaded with 5 µM (5-(6) Carboxyfluorescein diacetate Succinimidyl Ester “mixed isomer” (CFSE); InvitrogenTM) before being distributed to specific cell cultures. One µL (5 mM) CFSE was dissolved in 100 µL PBS, mixed thoroughly with B cells (10^6^ cells) in 900 µL complete RPMI-1640 medium, vortexed, and then incubated for 5min at RT protected from light. Then, 10 mL of 5% FCS-PBS were added, and the cells were centrifuged (10 min, 4 °C, and 300× *g*). This step was repeated twice to wash away the excess stain. We evaluated the cell recovery using trypan blue staining and a light microscope. After the specified stimulation period, we harvested and centrifuged the cell cultures and discarded the supernatants. The cells in co-cultures and triple cultures were stained with anti-CD4-APC to exclude the undesired Th/Treg cells from evaluation, and 7AAD was used for dead-cell exclusion.

### 4.6. ELISA

We used a home-developed ELISA protocol to determine the amounts of secreted IgG in the supernatants of cell cultures. Medium binding plates were coated overnight at 4 °C with 50 µL/well (3 µg/mL) rat anti-human IgG Fc (BioLegend). The plates were washed three times with 200 µL PBS-Tween20 (0.05%), blocked for 2 h with 100 µL blocking buffer (5% BSA-PBS-Tween20, 0.05%), and then washed as mentioned earlier. Afterward, the samples and a dilution series of standard IgG solution were added in triplicate (50 µL) and incubated for 2 h. The plates were then washed three times with washing buffer, and 50 µL goat anti-human IgG(H+L)-HRP (Southern Biotech, Birmingham, AL, USA) was added for 2 h at a dilution of 1:17,000, followed by three times washing. The signals were developed with 50 µL 3,3′,5,5′-Tetramethylbenzidine (TMB) (BioLegend), which was stopped after 10 min using 2N H_2_SO_4_. Finally, the absorbance was evaluated at 450 nm and 620 nm via a Multiskan™ Sky Microplate Spectrophotometer (Thermo Fisher Scientific, Waltham, MA, USA).

### 4.7. Statistical Analysis

The data were checked for normality before analysis. Accordingly, we performed an ordinary two-way ANOVA, followed by an uncorrected Fisher’s LSD test or Kruskal–Wallis test with an uncorrected Dunn’s test using Prism 9 for Windows version 9.0.0 (121). All data were represented as the mean ± SEM, and the difference was considered significant if *p* < 0.05.

## Figures and Tables

**Table 1:** Clinical and demographic data for RA patients.

	RA (*n* = 25)	Reference Value (RV)
Age (y; mean [range])	60.4 (24–85)	-
M/F (N)	1/24	-
Disease duration (y; mean [range])	11.07 (2–27)	-
Treatments:		
Methotrexate	12	-
Methotrexate + folic acid	1	-
Methotrexate + Medrol	6	-
Hydroxychloroquin + Medrol	1	-
Methotrexate + Medrol + tofacitinib	1	-
Cimzia	1	-
Somatostatin Analogues + Cimzia	1	-
Leflunomide	2	-
CRP (mg/L; mean [range])	11.07 (0.58–46)	<8 mg/L
DAS28 [ESR-based] (mean [range])	4.19 (1.6–5.58)	
aCCP (IU/mL)		<20 EU/mL
aCCP^−^	*n* = 5	
aCCP^+^ (mean [range])	1623.16 (70.15–3200)	
RF(IU/mL)		<20 IU/mL
RF^−^	*n* = 2	
RF^+^ (mean [range])	186.1 (12–650)	

Abbreviations: M/F: male/female, CRP: C-reactive proteins, DAS28: disease activity score using 28 joint counts, ESR; erythrocyte sedimentation rate, aCCP: anti-cyclic citrullinated peptide antibody, RF: rheumatoid factor mg/L: milligrams per liter, EU/mL: endotoxin units per milliliter, IU/mL: international units per milliliter.

## Data Availability

The data will be available upon request.

## Supplementary Materials

The following supporting information can be downloaded at https://www.mdpi.com/article/10.3390/ijms251810190/s1.

## Abbreviations

7AAD	7-Aminoactinomycin D
ACPA	Anti-citrullinated protein antibody
Bcl 6	B-cell lymphoma 6
BLIMP1	B-lymphocyte-induced maturation protein 1
CFSE	Carboxyfluorescein diacetate Succinimidyl ester “mixed isomer”
CTLA4	Cytotoxic T-lymphocyte-associated protein 4
FMO	Fluorescent minus one
Foxp3	forkhead box P3
HRP	Horseradish peroxidase
TMB	3,3′,5,5′-Tetramethylbenzidine
ICOS	T-cell inducible co-stimulator
MACS	Magnetic activating cells sorting
MB	Memory B cells
PB	Plasma blast cells
PBMCs	Peripheral blood mononuclear cells
PC	Plasma B cells
PD-1	Programmed cell death protein 1
PMA	Phorbol 12-myristate 13-acetate
RANKL	Receptor activator of nuclear factor kappa beta (NF-kβ) ligand
RF	Rheumatoid factor
SEB	Staphylococcus enterotoxin B
Tfh	follicular T helper cells
Tph	Peripheral T helper cells
