# Supplementary material for: Helper T Cells are Hyperactive and Contribute to the Dysregulation of Antibody Production in Patients with Rheumatoid Arthritis
Source: Int J Mol Sci. 2024 Sep 23;25(18):10190. doi: 10.3390/ijms251810190 (PMC11431999; doi:10.3390/ijms251810190)
Supplement: Supplementary file 1 [file ijms-25-10190-s001.zip › ijms-3199104-supplementary.pdf]

Supplementary Figures

Supplementary Figure S1

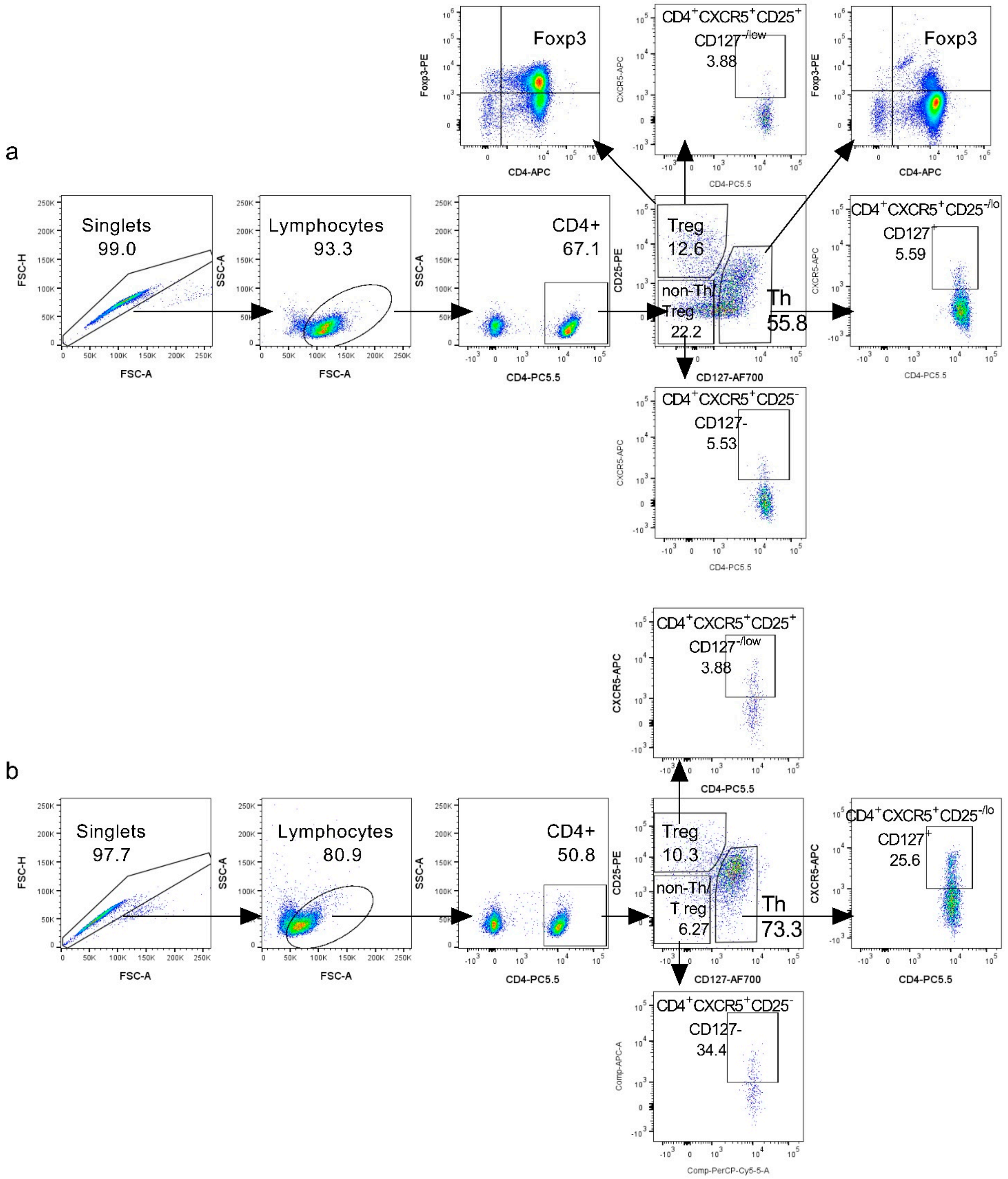

Supplementary Figures

Figure S1. A gating strategy for monitoring the frequencies of various CD4+ T cell subsets among lymphocytes from HCs (a) and RA patients (b).

Supplementary Figure S2

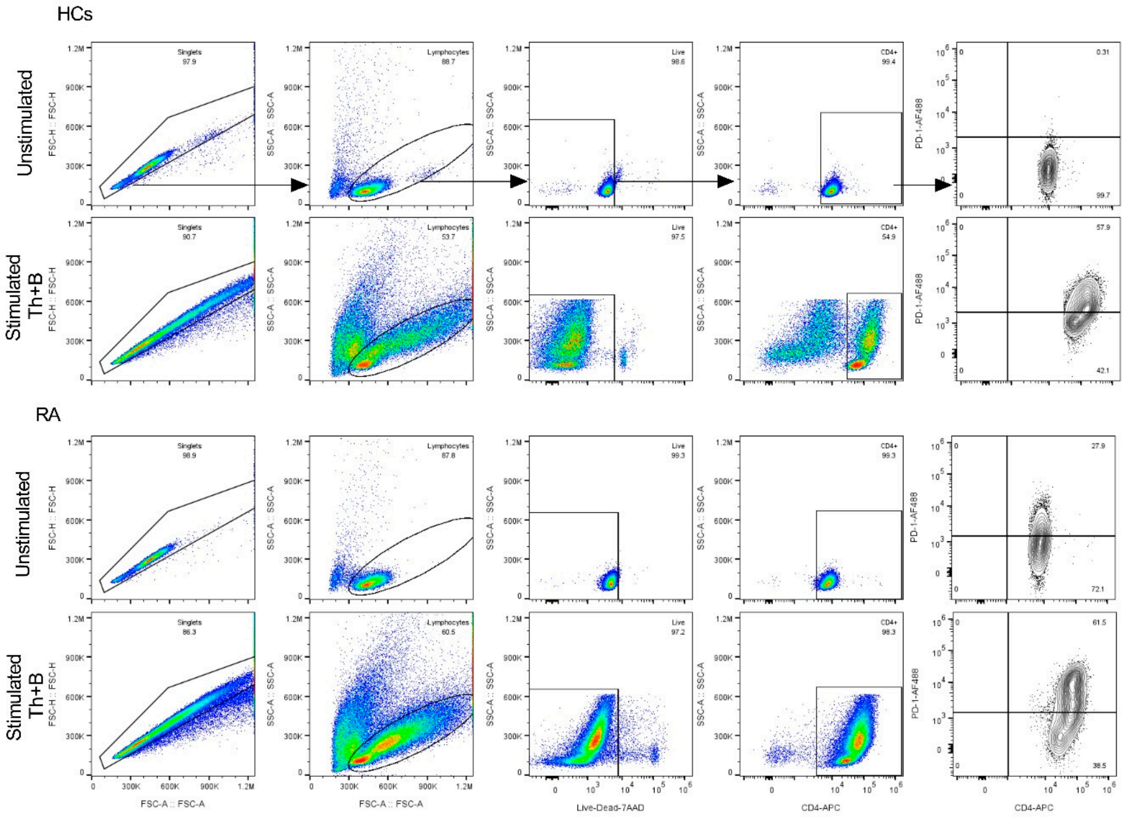

Figure S2. A full-gating strategy for detecting PD-1 expression among CD4+ Th cells from HC and RA patients' samples before and after stimulation with SEB superantigen in the presence of B cells.

Supplementary Figures

Supplementary Figure S3

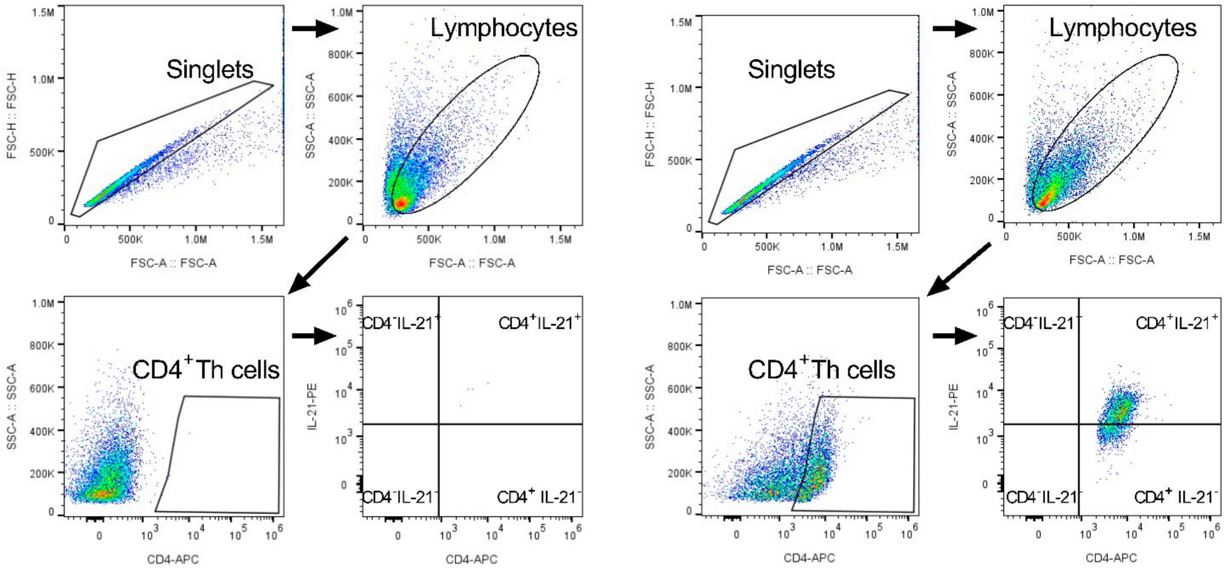

Figure S3. Gating strategy for characterizing IL-21 expressing CD4⁺ Th cells.

Supplementary Figures

Supplementary Figure S4

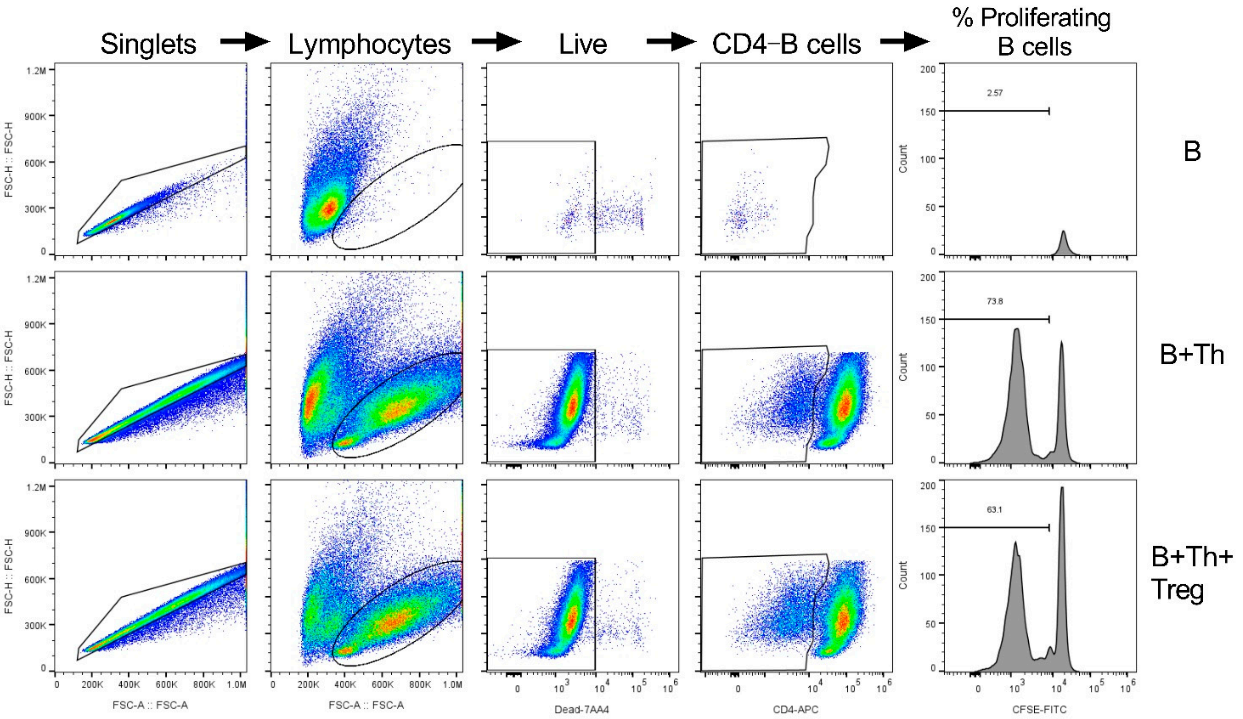

Figure S4. A gating strategy for the evaluation of B cell proliferation in SEB-stimulated B cell monocultures, co-cultures (Th+B), and in triple cultures (Th+B+Treg).

Supplementary Figures

Supplementary Figure S5

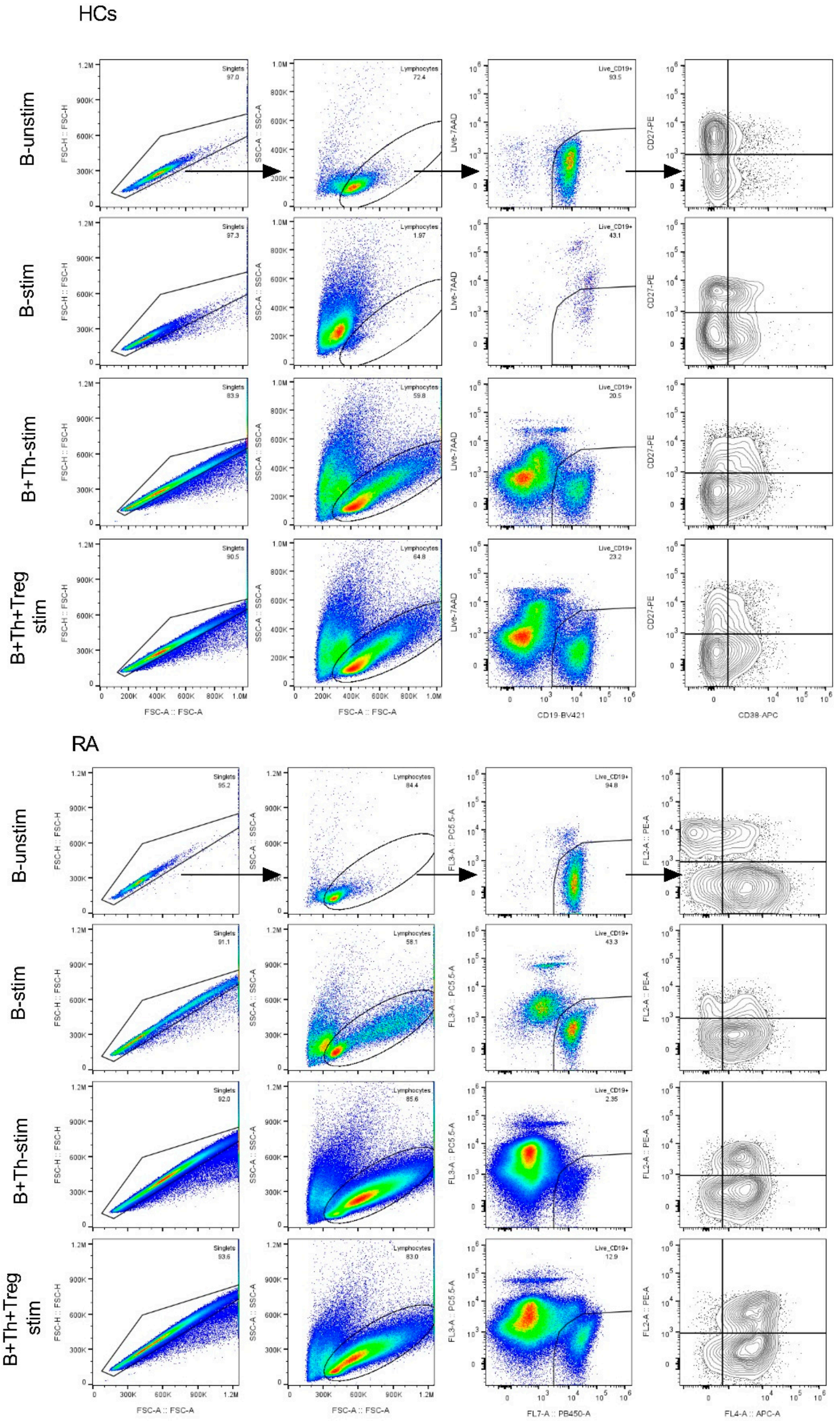

Supplementary Figures

Figure S5. 2. A full-gating strategy for evaluating B cell subsets on unstimulated B cells, SEB Stimulated B cell monoculture, B+Th cell, and B+Th+Treg cell cultures from HCs (Top) and RA samples (bottom).

Supplementary Figures

Supplementary Table S1

Supplementary Table S1 List of antibody conjugates and Isotype Controls

Antibody	Clone	Cat No.	Supplier
PerCp/Cyanine5.5 anti-human CD4	RPA-T4	300530	Bio-Legend
Phycoerythrin anti-human CD25	BC96	302606	Bio-Legend
Alexa Fluor®700 anti-human CD127	A019D5	351344	Bio-Legend
Phycoerythrin anti-human Foxp3	QA18A03	364704	Bio-Legend
Brilliant Violet 421™ anti-human CD19	HIB19	302234	Bio-Legend
Phycoerythrin anti-human CD27	O323	302808	Bio-Legend
Allophycocyanin anti-human CD38	HIT2	21270386	ImmunoTool
Phycoerythrin anti-human IL-21	3A3-N2	513004	Bio-Legend
Allophycocyanin anti-human CD4	MEM-241	21270046	ImmunoTools
Alexa Fluor® 488 anti-human CD279 (PD1)	NAT105	367408	Bio-Legend
**Isotype**	**Clone**	**Cat No.**	**Supplier**
Alexa Fluor® 700 Mouse IgG1, k Isotype Ctrl	MOPC-21	400143	Bio-Legend
PerCP/Cyanine5.5 Mouse IgG1, k Isotype Ctrl	MOPC-21	400149	Bio-Legend
PE Mouse IgG1, k Isotype Ctrl	MOPC-21	400111	Bio-Legend
Brilliant Violet421™ Mouse IgG1, k Isotype Ctrl	MOPC-21	400157	Bio-Legend
Alexa Fluor® 488 Mouse IgG1, k Isotype Ctrl	MOPC-21	400134	Bio-Legend
Mouse IgG1 Kappa Isotype control, eBioscience™ APC	P3.6.2.8.1	1943772	eBioscience
